# Sleep Apnea

**DOI:** 10.1155/2012/308978

**Published:** 2012-06-13

**Authors:** Manos Alchanatis, James MacFarlane, Sofia Schiza

**Affiliations:** ^1^Sleep Disorders Unit, 1st Department of Respiratory Medicine, National and Kapodistrian University of Athens, Greece; ^2^Med Sleep (Network of Clinics), University of Toronto, Toronto, Canada; ^3^Sleep Disorders Unit, Department of Thoracic Medicine, University of Crete, Greece

The obstructive sleep apnea syndrome (OSAS), a form of periodic breathing, is highly prevalent disorder that affects at least 4% of adult male population and 2% of females and is associated with repetitive episodes of complete (apnea) or partial (hypopnea) occlusion of upper airway during sleep. These episodes result, among others, in loud snoring and in daytime symptoms such as excessive sleepiness, morning headache, irritability, cognitive impairment and lead to reduced quality of life. Varying degrees of transient oxygen desaturation follow the upper airway occlusion, and it is a common pathway for the development of several cardiovascular complications. Central Sleep Apnea (CSA) is another form of periodic breathing characterized by lack of respiratory effort during sleep and frequently coexists with OSA. One major underlying mechanism of CSA is unstable ventilatory drive during sleep. Treatment options for sleep apnea include continuous positive airway pressure (CPAP), oral appliances, and weight loss.

In this special issue on sleep-related breathing disorders a number of distinguished scientists report on the following: the association between the duration of sleep and cardiovascular and metabolic comorbidities of patients with OSAS, the influence of age on clinical characteristics and polysomnographic findings of patients with OSAS in Greek population, the effect of weight loss by behavioural, pharmacological, or surgical approaches in the management of OSAS, the mechanisms that may trigger cognitive decline in OSA patients, the evaluation of two different mandibular advance appliances and the application of sclerosant agents for treatment of snoring, the presence of Cheyne-Stokes respiration (CSR), a form of central sleep apnea, in patients with radiologically-proven first-ever lacunar stroke, the implication of age in the pathogenetic link between sleep apnea and plasma homocysteine levels.

All the above help us to better understand some mechanisms involved in pathogenesis, consequences, and treatment of sleep apnea syndrome and snoring, and clearly this special issue is a great contributor to our knowledge.

*Manos Alchanatis* *Manos Alchanatis* *James MacFarlane* *James MacFarlane* *Sofia Schiza* *Sofia Schiza*

